# *GPR155* Serves as a Predictive Biomarker for Hematogenous Metastasis in Patients with Gastric Cancer

**DOI:** 10.1038/srep42089

**Published:** 2017-02-06

**Authors:** Dai Shimizu, Mitsuro Kanda, Haruyoshi Tanaka, Daisuke Kobayashi, Chie Tanaka, Masamichi Hayashi, Naoki Iwata, Yukiko Niwa, Hideki Takami, Suguru Yamada, Tsutomu Fujii, Goro Nakayama, Michitaka Fujiwara, Yasuhiro Kodera

**Affiliations:** 1 Department of Gastroenterological Surgery (Surgery II), Nagoya University Graduate School of Medicine, Nagoya, Japan

## Abstract

The prognosis of patients with gastric cancer (GC) with hematogenous metastasis is dismal. Identification of biomarkers specific for hematogenous metastasis is required to develop personalized treatments that improve patients’ outcomes. Global expression profiling of GC tissues with synchronous hepatic metastasis without metastasis to the peritoneal cavity or distant lymph nodes was conducted using next-generation sequencing and identified the G protein-coupled receptor 155 (*GPR155*) as a candidate biomarker. *GPR155* transcription was suppressed in GC cell lines compared with a nontumorigenic cell line. DNA methylation of the *GPR155* promoter region was not detected, albeit 20% of GC cell lines harbored copy number loss at GPR155 locus. The expression levels of *GPR155* mRNA correlated inversely with those of *TWIST1* and *WNT5B*. Inhibition of *GPR155* expression increased the levels of *p-ERK1/2* and *p-STAT1*, significantly increased cell proliferation, and increased the invasiveness of a GC cell lines. *GPR155* mRNA levels in GC clinical samples correlated with hematogenous metastasis and recurrence. Multivariate analysis revealed that reduced expression of *GPR155* mRNA was an independent predictive marker of hematogenous metastasis. *GPR155* may represent a biomarker for diagnosing and predicting hematogenous metastasis of GC.

Gastric cancer (GC) is the fourth most common cancer and the third most frequent cause of cancer-related death worldwide (723,000 deaths in 2012)^1^. Despite improvements in the diagnosis of GC at an early stage and the availability of new anticancer agents, the 5-year survival rate of patients with advanced GC is only 25–30%^2^. Increased efforts to eradicate *Helicobacter pylori* in Asian countries are expected to reduce the incidence of GC in the middle or lower gastric tract^3^,^4^. In contrast, the incidence of the intestinal type of differentiated GC located in the upper stomach or esophagogastric junction will likely increase worldwide^5^. Such tumors have relatively high incidence of hematogenous metastasis than GC in the middle or lower gastric tract. Therefore, effective management of hematogenous metastasis of GC is raised as an important clinical issue that must be resolved urgently. For the first important step to achieve this goal, discovery of sensitive and specific biomarkers for hematogenous metastasis is required to identify patients at high risk.

GC metastasizes through three dominant processes; hematogenous, lymphatic and direct dissemination from the serosal surface. Among them, hematogenous metastasis requires a distinctive multistep process involving vascular invasion, detachment from a primary site, survival of tumor cells in hypoxic portal blood, tissue engraftment, evasion of the hepatic immune system, and colonization^6^,^7^. The application of next-generation sequencing technology reveals that an underlying molecular signature of a tumor cell’s ability to metastasize varies according to the metastatic process and target organs^8^,^9^,^10^,^11^. We hypothesized that metastasis from primary GC via hematogenous route employs a specific mechanism that can be exploited to identify specific biomarkers for hematogenous metastasis of GC.

Therefore, in the present study, we conducted global expression profiling according to the metastatic route to identify molecules specific for hematogenous metastasis and show that the G protein-coupled receptor 155 (*GPR155*) may serve as a candidate biomarker.

## Results

### Identification of Candidate Marker

We sought candidate markers according to the following sieving criteria (Fig. S1). Firstly, this time we targeted molecules with decreased expression levels in GC tissues compared with the corresponding noncancerous mucosal tissues. Secondly, we focused on the ability which had been already got in the primary lesion to metastasize via hematogenous route. Then, we imposed the condition that there were no differences in gene expression in the primary lesion and hepatic metastases. As a result, the transcriptome analysis identified 21 candidate genes related to hematogenous metastasis (Table 1). Among them, the largest decrease in expression in GC tissues compared with noncancerous mucosae was that of *GPR155*. G protein-coupled receptor (GPCR) family have been reported to contribute to tumor progression through interactions with cancer-related signaling pathways. However, there are no reports to our knowledge of an association of *GPR155* with GC. Therefore, we focused on *GPR155* in this study.

### Expression of GPR155 and Potentially Interacting Molecules

**Figure 1:**
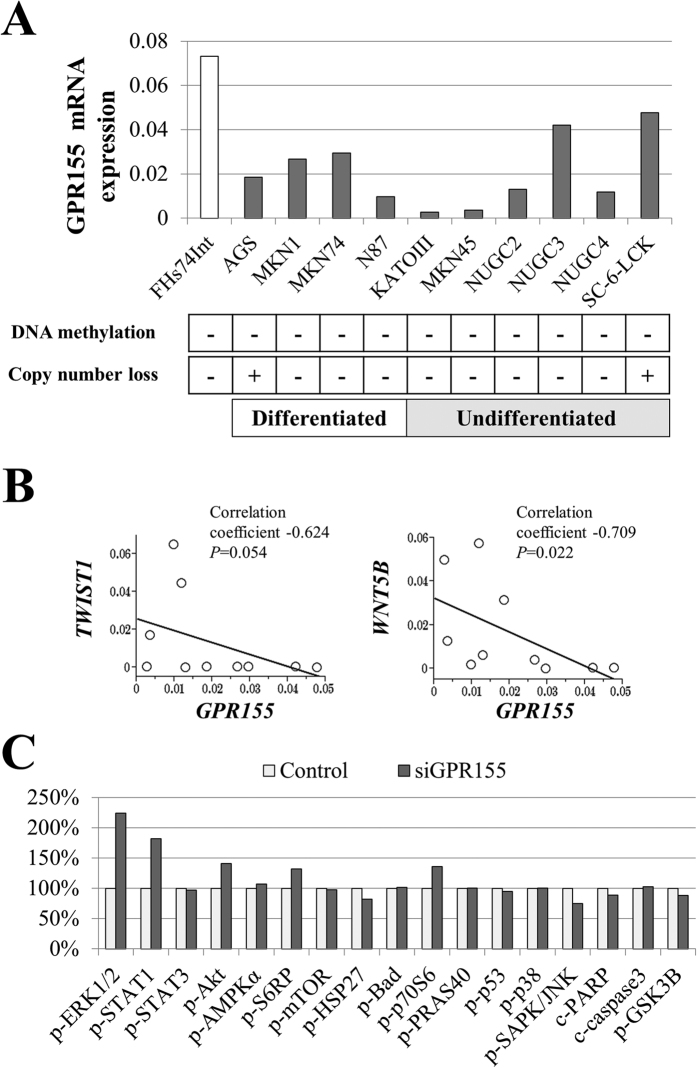
Analysis of *GPR155* expression and identification of candidate molecules that interact with *GPR155*: assays used indicate downstream effects. (**A**) Reduced expression of *GPR155* mRNA was detected in all GC cell lines compared with that of FHs74Int. Copy number loss was detected in AGS and SC-6-JCK cells. Methylation of the promoter region of *GPR155* was not detected. (**B**) PCR array analysis showing that the expression level of *GPR155* mRNA inversely correlated with those of *TWIST1* and *WNT5B* mRNAs. (**C**) Cell signaling pathway analysis of AGS cells indicated that inhibition of *GPR155* mRNA increased the levels of *p-ERK1/2* and *p-STAT1*.

Reduced expression of *GPR155* mRNA was detected in all GC cell lines compared with FHs74Int (Fig. 1A). MKN1, MKN45 and N87 are cell lines which were established from hepatic metastasis. *GPR155* mRNA expression was strongly suppressed in MKN45 and N87 cells. Little is known about cancer-related molecules that interact with *GPR155*^12^. To address this gap in our knowledge, we conducted PCR array analysis and found that the expression level of *GPR155* mRNA correlated inversely with those of *TWIST1* and *WNT5B* mRNAs (Fig. 1B and Fig. S2A). The expression levels of *GPR155, TWIST1,* and *WNT5B* were not related to the state of differentiation of GC cell lines (Fig. S2A). Pathway analysis of AGS cells indicated that inhibition of *GPR155* mRNA expression increased the levels of *p-ERK1/2* and *p-STAT1* by >50% (Fig. 1C and Fig. S3).

### Analysis of Mechanisms that Inhibit GPR155 Expression

We conducted bisulfite sequencing to investigate whether DNA methylation inhibits *GPR155* transcription. Methylation of cytosine residues within the promoter region of *GPR155* was not detected in the GC cell lines or FHs74Int, (Fig. 1A and Fig. S2B). TaqMan Copy Number Assays for identification of possible regulatory mechanisms of *GPR155* expression other than DNA methylation detected copy number loss in AGS and SC-6-JCK cells but not in FHs74Int cells (Fig. 1A).

### Effects of GPR155 Knockdown on GC Cell Activities

**Figure 2:**
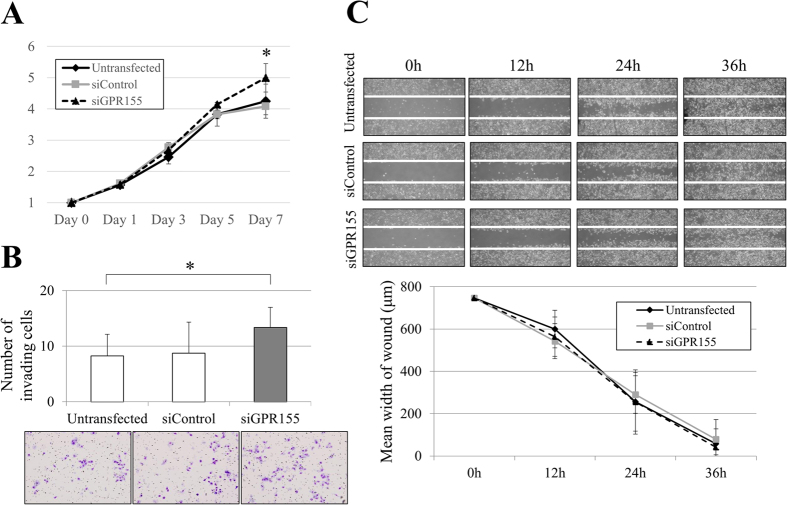
Phenotypes of AGS cells transfected with si*GPR155*. (**A,B**) Knockdown of *GPR155* expression increased cell proliferation and invasiveness. (**C**) There was no significant change in cell migration.

We inhibited *GPR155* mRNA expression by transfecting AGS and MKN1 cells with si*GPR155* (Fig. S2C) to determine the influence of *GPR155* on cell phenotype and the contribution of *GPR155* to untransfected AGS and MKN1 those expressed relatively high levels of *GPR155*. We evaluated the proliferation, migration, and invasion of AGS and MKN1 cells. *GPR155* knockdown significantly increased cell proliferation on day 7 compared with untransfected and siControl cells both in AGS and MKN1 cells (Fig. 2A and Fig. S4A). Moreover, the number GC cells invading the matrigel were increased in cells transfected with si*GPR155* both in AGS and MKN1 cells (Fig. 2B and Fig. S4B). There was no significant change in migration of AGS cells when *GPR155* expression was inhibited (Fig. 2C), whereas the migration ability of MKN1 cells was increased by knockdown of *GPR155* expression (Fig. S4C).

### Diagnostic Performance of GPR155 Expression Levels

**Figure 3:**
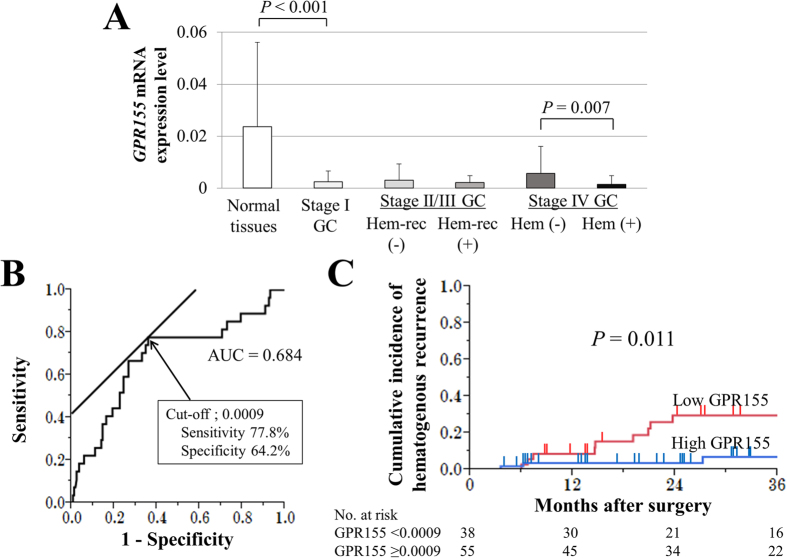
Expression of *GPR155* in clinical samples, and the cumulative incidence of hematogenous recurrence in patients with Stage II/II GC. (**A**) Significantly lower levels of *GPR155* mRNA were detected in primary GC tissues compared with the corresponding noncancerous mucosal tissues. Patients with Stage IV GC with synchronous hematogenous metastasis had lower expression levels of *GPR155* mRNA compared with those of patients without hematogenous metastasis. (**B**) The optimal cut-off value of *GPR155* expression was determined at 0.0009. (**C**) The cumulative incidence of hematogenous recurrence was significantly higher in the low *GPR155* group patients with Stage II/III GC. *Abbreviations*: Hem-rec; hematogenous recurrence, Hem; synchronous hematogenous metastasis.

Primary GC tissues expressed significantly lower levels of *GPR155* mRNA compared with the corresponding noncancerous mucosal tissues (Fig. 3A). Even in tissues of patients with Stage I GC, *GPR155* mRNA expression was reduced by approximately 10-fold. *GPR155* mRNA levels in GC tissues were significantly lower in stage IV patients with synchronous hematogenous metastasis compared with those without synchronous hematogenous metastasis. By looking into patients with stage II/III GC, *GPR155* mRNA levels were lower in GC tissues from patients with metachronous hematogenous metastasis compared to those from patients without metachronous hematogenous metastasis, though the difference was not statistically significant (Fig. 3A).

The optimal cut-off value of the *GPR155* mRNA level in GC tissues was determined at 0.0009 having a modest correlation (AUC = 0.684) with synchronous and metachronous hematogenous metastasis by the receiver operating characteristic curve analysis in all 200 GC patients (Fig. 3B). Using this cut-off value, we stratified patients into low *GPR155 (GPR155* < 0.0009) and high *GPR155 (GPR155* ≥ 0.0009) groups. Analysis of the association between the *GPR155* mRNA level and clinicopathological factors shows that low *GPR155* mRNA levels in GC tissue significantly associated with age ≥ 65 years, male sex, macroscopic type (other than Borrmann type4/5), low T stage, differentiation, expansive growth type, and synchronous hematogenous metastasis (liver n = 11, lung n = 2, bone n = 1 and brain n = 1) (Table 2).

**Figure 4:**
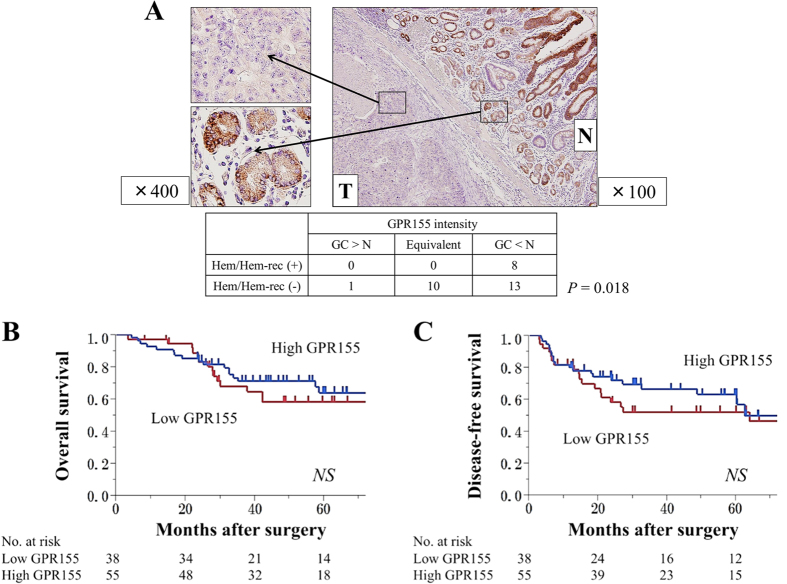
IHC analysis of *GPR155* expression in tissues of representative patients with GC and the survival curve of patients with Stage II/III GC. (**A**) *GPR155* staining intensity of GC tissues was generally low compared with the corresponding noncancerous mucosae. *GPR155* staining intensity correlated significantly with synchronous and metachronous hematogenous metastasis. (**B**) There was not significant difference in overall survival between low *GPR155* and high *GPR155* groups with Stage II/III GC. (**C**) There was no significant difference in disease-free survival between the low *GPR155* and high *GPR155* groups with Stage II/III GC. *Abbreviations*: T; tumor tissue, N; non-cancerous component, GC; primary gastric cancer tissue, Hem/Hem-rec; hematogenous metastasis/recurrence.

The cumulative incidence of hematogenous recurrence was significantly higher in the low *GPR155* group (Fig. 3C), while the incidence of postoperative adjuvant chemotherapy (S-1 monotherapy) was not different between two groups (Table 2). Hematogenous recurrences were found at the liver (n = 9), bone (n = 1), brain (n = 1) and ovary (n = 1). Otherwise, there was no significant difference in overall survival between the two groups in all Stages (5-year survival rates, 53% and 59%, respectively). Similarly, in patients with Stage II/III GC with or without hematogenous recurrence with significant differences in *GPR155* mRNA levels, there were no significant differences in overall survival (Fig. 4B) and disease-free survival (Fig. 4C) between the low and high *GPR155* groups. Multivariate analysis identified low *GPR155* mRNA expression levels in GC tissue as an independent predictive factor of synchronous hematogenous metastasis and metachronous hematogenous metastasis after curative gastrectomy (hazard ratio, 5.38; *P* = 0.001), together with CEA > 5 ng/ml, vessel invasion, and expansive growth (Table 3).

### Association between Hematogenous Metastasis and *in situ* Expression of GPR155 Protein

**Figure 5:**
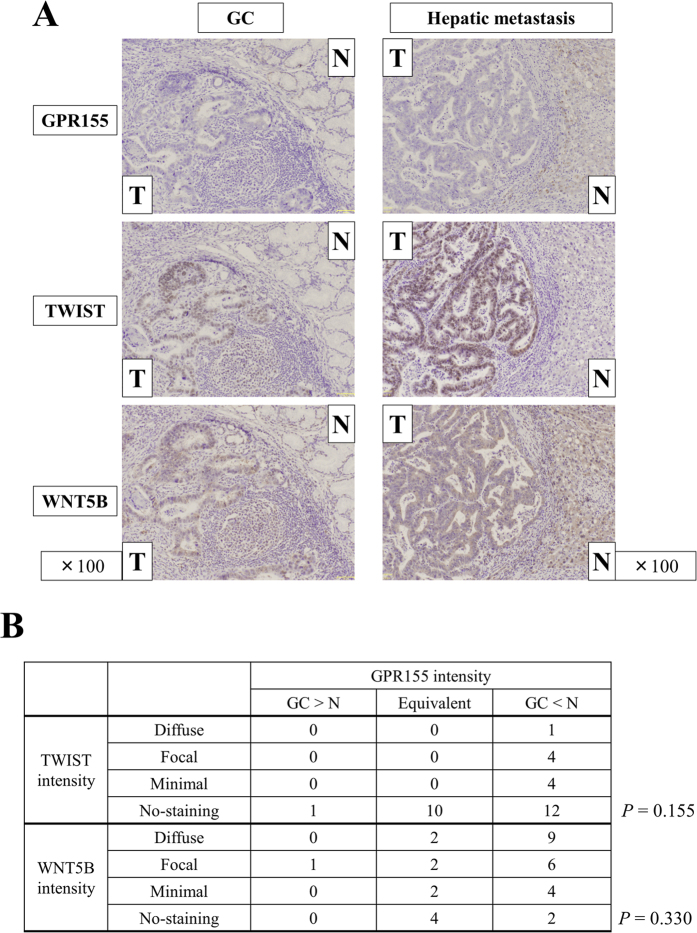
Immunohistochemical staining for GPR155, TWIST and WNT5B proteins. (**A**) Results in a representative case using tissues from primary GC and hepatic metastasis. (**B**) Correlations in staining patterns between GPR155, TWIST and WNT5B. *Abbreviations*: T; tumor tissue, N; non-cancerous component, GC; primary gastric cancer tissue.

We performed Immunohistochemical (IHC) to verify whether the expression of *GPR155* was significant for diagnosing and predicting hematogenous metastasis. *GPR155* staining intensity in GC tissues was lower compared with the corresponding noncancerous mucosae in most cases. Moreover, lower levels of *GPR155* correlated significantly with synchronous hematogenous metastasis and metachronous hematogenous metastasis that occurred after curative gastrectomy (Fig. 4A). Strong (focal or diffuse) staining TWIST and WNT5B tended to be found in patients with suppressed GPR155 at GC components (Fig. 5).

## Discussion

We performed transcriptome analysis to identify hematogenous metastasis-specific biomarkers of GC. Among candidate molecules identified by our transcriptome analysis, the largest difference in expression levels between primary GC tissue and the corresponding noncancerous mucosal was exhibited by *GPR155* mRNA. In contrast, *GPR155* mRNA was expressed at equivalent levels in primary GC and hepatic metastatic lesions. These findings indicate that decreased expression of *GPR155* may reflect the inherent metastatic potential of GC cells in the primary tumor that was not acquired during metastasis via hematogenous route.

*GPR155*, which resides on chromosome 2q31.1, comprises eighteen exons and encodes a 97 kDa protein. *GPR155* is a member of the seven-transmembrane domain of the GPCR family^13^. Ligand binding activates the guanine nucleotide exchange factor activity of GPCRs that exchange GDP for GTP on its associated G protein. The Gα subunit bound to GTP dissociates from the Gβ and Gγ subunits to activate intracellular signaling proteins or target proteins directly. Limited information is available on the ligands for GPCRs at present. Huang XP, *et al*. detected some ligands for other GPCR families, GPR68 and GPR65^14^. GPCRs mediate diverse physiological processes such as the visual sensing, immune function, cell proliferation, and tumor metastasis^15^,^16^. It is therefore not surprising GPCRs represent 30–50% of the targets of currently marketed therapeutic drugs^17^,^18^,^19^,^20^. *GPR155* is a unique member of GPCRs and there have been only a few reports on involvement in malignancies, such as follicular type papillary thyroid carcinoma and colorectal cancer^21^,^22^. In those earlier studies, *GPR155* was listed in the results of microarray or proteomic analysis, and no data on the function and clinical significance of *GPR155* was presented.

In the present study, qRT-PCR revealed varying levels of *GPR155* mRNA expression level in GC cell lines independent of their differentiation phenotypes. In all GC cell lines tested here, *GPR155* mRNA expression was reduced compared with that of the human intestinal epithelial cell line FHs74Int. To seek for the regulatory mechanisms of *GPR155* transcription, DNA methylation of the CpG island of a promoter region prevents transcription^23^, and the promoter region of *GPR155* harbors a CpG island; however, we were unable to detect promoter methylation in the genomes of any of the GC cell lines studied here. Therefore, we investigated copy number loss as a possible secondary suppression mechanism. Copy number loss was detected in 20% of the GC cell lines, which suggests the possibility that copy number loss may contribute to the suppression of *GPR155* mRNA expression. However, histone methylation, micro-RNAs, transcription suppressors and RNA editing may contribute to the suppression of *GRP155* expression, and therefore, further investigations are mandatory to elucidate the regulatory mechanisms of *GPR155* expression^24^,^25^.

As little evidence is available on the oncological roles of *GPR155*, we performed PCR array and cell signaling pathway analyses to identify cancer-related molecules that potentially interact with *GPR155*. Although statistical power was not strong due to number of analyzed cell lines, we detected an inverse correlation between *GPR155* mRNA expression and those of *TWIST1* and *WNT5B* mRNA. *TWIST1* promotes metastasis through its effects on the epithelial-mesenchymal transition and promotes the formation of distant metastasis in GC^26^,^27^,^28^,^29^. *WNT5B* is associated with tumor formation and malignant transformation in GC, breast cancer, and squamous cell carcinoma of the head and neck^30^,^31^,^32^,^33^. The correlation between the expression of *GPR155* and these molecules suggests that the suppression of *GPR155* expression interferes with oncogenic signaling pathways. To test this hypothesis, we investigated the downstream effect of inhibiting *GPR155* expression on signaling transduction pathways that mediate cell proliferation. We detected increased *p-ERK1/2* and *p-STAT1* compared with controls. Gα subunit are divided into several subtypes, in which there are two major subtypes, Gsα activating adenylyl cyclase (AC) and Giα suppressing AC^34^. To date, the type of Gα subunit coupling to GPR155 has not been specified. Our result suggests that the Gα subunit coupling to *GPR155* may be Giα subunit which suppresses AC.

We selected two GC cell lines expressing relatively high *GPR155* mRNA from differentiated type for assays of cell phenotype, AGS having copy number loss and MKN1 established from hepatic metastasis. In both GC cell lines, *GPR155* knockdown led to significant increases in cell proliferation and invasion, indicating that *GPR155* has downstream effect to cancer-related signaling pathways and therefore acts as a tumor suppressor. Studies on the effect of *GPR155* knockdown on apoptosis and the cell cycle may reveal how *GPR155* affects cell proliferation. Using cells cultured under hypoxic conditions or in suspension may reveal how *GPR155* participates in the mechanism of hematogenous metastasis. Further, mouse xenograft models might provide information on tissue engraftment, invasion, and colonization via hematogenous route.

The most important finding of the present study was that *GPR155* expression demonstrated high diagnostic and predictive performance for hematogenous metastasis of GC. *GPR155* mRNA levels in GC tissues were significantly decreased in all stages, including Stage I, compared with the corresponding normal mucosa. While *GPR155* mRNA expression significantly decreased in GC tissue, it decreased further in patients with synchronous hematogenous metastasis compared to those without. These findings indicate that *GPR155* represents a hematogenous metastasis-specific biomarker for GC. Low differentiation, serosal invasion, diffuse type, young age and infiltrating growth are well-known risk factors for peritoneal dissemination^35^,^36^,^37^,^38^, and vascular invasion, advanced age, differentiation, Borrmann type 1 or 2, expansive growth have been reported to be risk factors for hepatic metastasis, most frequent hematogenous metastasis of GC^35^,^36^,^39^,^40^. The clinicopathological factors associated with reduced *GPR155* expression reported here conflict with known risk factors for peritoneal dissemination and are, however, associated with known risk factors for hepatic metastasis. Moreover, multivariate analysis revealed that suppression of *GPR155* mRNA expression was an independent prognostic factor for synchronous hematogenous metastasis and metachronous hematogenous metastasis that occurred after curative gastrectomy. These clinicopathological analyses support the hypothesis that *GPR155* is specifically associated with hematogenous metastasis of GC and that detecting reduced levels of *GPR155* mRNA in primary GC tissue is useful for the diagnosis of synchronous hematogenous metastasis as well as for determining a patient’s risk of hematogenous recurrence after curative gastrectomy. On the other hand, lower expression of *GPR155* mRNA in GC tissues was not associated with overall or disease-free survival. Nevertheless, the cumulative incidence of hematogenous recurrence in patients with Stage II/III GC was significantly higher in the low *GPR155* group. This is rational result, because *GPR155* expression level is specific for hematogenous metastasis and survivals are significantly influenced by other metastatic patterns, peritoneal dissemination or distant lymph node metastasis.

Translating our results into clinical practice, physicians can stratify GC patients according to the risk for hematogenous metastasis by performing qRT-PCR or IHC analysis of biopsy or surgical specimens of primary tumor. For patients at high risk of hematogenous metastasis, appropriate management may be provided according to careful preoperative examinations, and postoperative surveillance focusing on hematogenous metastasis will facilitate early detection and therapeutic intervention. These measures will likely contribute to improve the outcomes of patients with GC.

This study has certain limitations. First, this study was limited by the relatively small sample size. The clinical significance and the cut-off value of *GPR155* expression level as a hematogenous metastasis-specific marker of GC should be evaluated in a larger patient cohort. Second, further analyses of putative interacting molecules indicated here are required to identify the molecular mechanisms underlying the biological activities of *GPR155* in patients with GC. Third, enforced expression of *GPR155* is required for further evaluation of the function of *GPR155* in GC. Finally, the mechanism of *GPR155* expression in GC remains to be identified.

Our results indicate that *GPR155* is a biomarker that is useful for the diagnosis and prediction of hematogenous metastasis of patients with GC.

## Methods

### Transcriptome Analysis

Surgically resected specimens of four patients with GC with synchronous hepatic metastasis without metastasis to the peritoneal cavity or distant lymph nodes were subjected to transcriptome analysis. Global expression profiling was conducted using the HiSeq System (Illumina, San Diego, CA, USA) to compare the expression levels of 57,751 genes in primary GC tissues, the corresponding noncancerous adjacent gastric mucosae, and hepatic metastases.

### Sample Collection

We used GC cell lines, which were obtained from the Japanese Collection of Research Bioresources Cell Bank (Osaka, Japan) as follows: MKN1, MKN45, MKN74, NUGC2, NUGC3, NUGC4, and SC-6-JCK. The GC cell lines AGS, KATOIII, and N87 were purchased from the American Type Culture Collection (ATCC) (Manassas, VA, USA). The human intestinal epithelial cell line FHs74Int (ATCC) served as a nontumorigenic control. Primary GC tissues and corresponding noncancerous mucosal tissues were collected from 200 patients who underwent gastrectomy at Nagoya University Hospital between 2001 and 2014. None of the patients underwent preoperative chemotherapy.

The methods were carried out in accordance with relevant guidelines. The study protocol was approved by the Medical Ethics Committee of the Nagoya University Hospital, protocol No. 2014–0043. Informed consent was obtained from all patients. Written informed consent for the use of clinical samples and data, as required by the Institutional Review Board, was obtained from all patients.

### Quantitative Real-Time Reverse-Transcription Polymerase Chain Reaction (qRT-PCR) and PCR Array Analysis

*GPR155* mRNA levels were determined using qRT-PCR. Total RNAs (10 μg) were extracted from GC cell lines, FHs74Int, and 200 pairs of clinical samples and were amplified using *GPR155*-specific primers (Table 4). Glyceraldehyde-3-phosphate dehydrogenase (GAPDH) mRNA (TaqMan, GAPDH control reagents, Applied Biosystems, Foster City, CA, USA) was quantified in each sample for standardization (Table 4). *GPR155* mRNA expression level was determined as the value of *GPR155* divided by that of GAPDH. The qRT-PCR protocol was performed as previously described^41^. To identify genes encoding putative *GPR155*-interacting proteins, we used the Human Epithelial to Mesenchymal Transition (EMT) RT2 Profiler PCR Array (Qiagen, Chatsworth, CA, USA)^42^.

### DNA Methylation Analysis and Copy Number Analysis

We used CpG Island Searcher software (http://cpgislands.usc.edu/)^43^,^44^ to detect predicted CpG islands in the *GPR155* promoter region (chr2:174486637–174487426). Genomic DNAs of the cell lines were treated with bisulfite, and bisulfite sequence analysis was performed as previously described (Table 4)^45^. Using purified genomic DNA isolated from GC cell lines, DNA copy numbers were determined using TaqMan Copy Number Assays (Applied Biosystems). The assays were as follows: upstream (assay ID: Hs01092594_cn, 175351658 within exon 1), midstream (assay ID: Hs01971174_cn, 175335170 within exon 6), and downstream (assay ID: Mn00059996_cn, 73351855 overlaps intron 14 and exon 14). Copy number alteration in the *GPR155* locus were determined using CopyCaller™ Software (Life Technologies, Carlsbad, CA, USA)^46^.

### Inhibition of GPR155 Expression

Small interfering RNAs (siRNAs) specific for *GPR155* mRNA (si*GPR155*) (Table 4) (Hokkaido System Science, Sapporo, Japan) were used to transfect AGS cells. AccuTarget^TM^ Negative Control siRNA Fluorescein-labeled (Cosmo Bio Co. Ltd., Tokyo, Japan) served as a control nontargeting siRNA (siControl). AGS and MKN1 cells were seeded to grow to 60–80% confluence 24 h later and transfected with siRNAs using LipoTrust EX Oligo (Hokkaido System Science) as previously described^47^. After 72 h incubation following siRNA transfection, total RNAs and proteins were extracted. For assays of cell phenotype, the transfected cells were treated with EDTA-trypsin.

### Cell Signaling Pathway Analysis

We used the PathScan^®^ Intracellular Signaling Array Kit (Cell Signaling Technology, Beverly, MA, USA), according to the manufacturer’s protocol, to investigate the downstream effects of inhibiting *GPR155* expression on cell signaling pathways in AGS cells.

### Assays of Cell Phenotype

The proliferation of AGS and MKN1 cells transfected with si*GPR155* was evaluated using a Premix WST-1 Cell Proliferation Assay System (Takara Bio Inc., Kusatsu, Japan). Cells (5 × 10^3^ cells per well) were seeded into 96-well plates in DMEM supplemented with 2% FBS. The optical density of each well was measured in six replicates consist of six technical and two biological replicates on days 0, 1, 3, 5, and 7 after seeding. The ability of cells to invade Matrigel was determined using BioCoat Matrigel Invasion Chambers (BD Biosciences, Bedford, MA, USA) according to the manufacturer’s protocol. Cells (2.5 × 10^4^ cells per well) were seeded into the upper well of the chamber in serum-free DMEM, and DMEM supplemented with 10% FBS was supplied into the lower well. After an appropriate incubation time (AGS; 48 h, MKN1; 36 h), we used a light microscope to count the cells present on the surface of the membrane in eight randomly selected fields. Migration was determined using a published wound-healing assay^48^. Cells (2 × 10^4^ cells per well) were seeded into 12-well plates in serum-free DMEM using the ibidi Culture insert method (ibidi, Martinsried, Germany) to establish wound gaps of a defined width. After 24 h, the insert was removed, and the width of the wound was measured at 100-μm intervals (20 per well, 40× magnification).

### Immunohistochemical Analysis

IHC was performed to determine the localization of *GPR155, TWIST1* and *WNT5B* in 32 representative sections of well-preserved GC tissue and 4 representative sections of well-preserved hepatic metastasis. Sections were incubated for 1 h at room temperature with a rabbit polyclonal antibody raised against *GPR155* (sc-137511, Santa Cruz Biotechnology Inc., Dallas, TX, USA) diluted 1:100, a mouse monoclonal antibody raised against *TWIST* (ab175430, Abcam, Cambridge, UK) diluted 1:500 or a rabbit polyclonal antibody raised against *WNT5B* (ab115563, Abcam, Cambridge, UK), diluted 1:200 in Antibody Diluent (Dako, Carpenteria, CA, USA). Antigen-antibody complexes were visualized using liquid 3, 3’-diaminobenzidine (Nichirei, Tokyo, Japan) after a 2 min incubation. To avoid bias, specimens were randomized, coded, and then analyzed by two independent observers who were uninformed of the identities of the samples. Each observer evaluated all specimens at least twice within a given time interval to minimize intraobserver variation. Staining intensity of *GPR155* was categorized to three groups; increased (GC > non-cancerous component), equivalent and decreased (GC > non-cancerous component). Staining intensities of *TWIST* and *WNT5B* were scored by proportions of stained cells in GC component as follows; no-staining (0%), minimal (less than 30%), focal (30–60%) and diffuse (more than 60%)^49^.

### Statistical Analysis

Differences between the data of two groups were evaluated using the Mann–Whitney test. To divide patients into two groups, a cut-off value was determined according to the receiver operating characteristic curve analysis drawn by data from all 200 GC patients in stage I–IV GC including those with hematogenous metastasis. The χ^2^ test was used to analyze the significance of the association between *GPR155* mRNA expression levels and patients’ clinicopathological characteristics, the significance of the association between IHC intensity and the incidences of hematogenous metastasis, and correlations in IHC intensities. Survival rates were calculated using the Kaplan–Meier method, and the differences in survival curves were evaluated using the log-rank test. Predictive factors were evaluated using multivariate analysis. All statistical analyses were performed using JMP 10 software (SAS Institute Inc., Cary, NC). A p value < 0.05 was considered statistically significant.

## Additional Information

**How to cite this article:** Shimizu, D. *et al. GPR155* Serves as a Predictive Biomarker for Hematogenous Metastasis in Patients with Gastric Cancer. *Sci. Rep.* **7**, 42089; doi: 10.1038/srep42089 (2017).

**Publisher's note:** Springer Nature remains neutral with regard to jurisdictional claims in published maps and institutional affiliations.

## Supplementary Material

Supplementary Information

## Figures and Tables

**Table 1:** Candidate tumor suppressor genes downregulated in gastric cancer tissues of patients with hepatic metastasis.

Symbol	GC tissue/Normal		Full name	Location	Function	H-meta/GC tissue	
	Log_2_	*P*				Log_2_	*P*
GPR155	−4.43	<0.0001	G protein-coupled receptor 155	2q31.1	Multi-pass membrane protein	0.01	1.0000
MFSD4	−4.32	<0.0001	major facilitator superfamily domain containing 4	1q32.1	Membrane transporter	−0.69	0.4659
HRASLS2	−4.26	<0.0001	HRAS-like suppressor 2	11q12.3	Metabolic enzyme	−1.38	0.1086
SLC9A4	−3.95	<0.0001	solute carrier family 9, subfamily A, member 4	2q12.1	Signal transducer	−0.40	0.6221
ALDH3A1	−3.40	<0.0001	aldehyde dehydrogenase 3 family, member A1	17p11.2	Metabolic enzyme	−0.95	0.3085
DPT	−3.22	<0.0001	dermatopontin	1q12-q23	Extracellular matrix protein	−0.12	0.8893
POU2AF1	−2.93	<0.0001	POU class 2 associating factor 1	11q23.1	Transcription factor	−1.07	0.0575
IRF4	−2.92	<0.0001	interferon regulatory factor 4	6p25-p23	Transcription factor	−0.84	0.2034
ASAH2	−2.82	<0.0001	N-acylsphingosine amidohydrolase 2	10q11.21	Metabolic enzyme	0.09	1.0000
GSTA1	−2.76	0.0002	glutathione S-transferase alpha 1	6p12.1	Metabolic enzyme	1.17	0.0828
MZB1	−2.71	<0.0001	marginal zone B and B1 cell-specific protein	5q31.2	Immunoglobulin mediator	−1.19	0.1480
LIFR	−2.59	<0.0001	leukemia inhibitory factor receptor alpha	5p13-p12	Cytokine receptor	0.08	0.9059
AKR1B10	−2.57	0.0002	aldo-keto reductase family 1, member B10	7q33	Metabolic enzyme	0.22	0.8146
PAIP2B	−2.50	0.0002	poly (A) binding protein interacting protein 2B	2p13.3	Transcription factor	0.70	0.2801
PIM2	−2.43	<0.0001	Pim-2 proto-oncogene, serine/threonine kinase	Xp11.23	Protooncogene	−0.26	0.5942
GPAT3	−2.26	0.0002	glycerol-3-phosphate acyltransferase 3	4q21.23	Metabolic enzyme	1.13	0.0679
XYLT2	−2.09	<0.0001	xylosyltransferase II	17q21.33	Metabolic enzyme	−0.15	0.7747
METTL7A	−2.09	0.0002	methyltransferase like 7 A	12q13.12	Methyltransferase	−0.28	0.6188
FAM46C	−2.03	<0.0001	family with sequence similarity 46, member C	1p12	Translational factor	−0.28	0.5389
IL7R	−2.02	0.0002	interleukin 7 receptor	5p13	Cytokine receptor	−0.34	0.5839
BTG2	−1.83	0.0001	BTG family, member 2	1q32	Transcription factor	0.78	0.1001

*Abbreviations: GC tissue*: primary gastric cancer tissue, *Normal*: corresponding adjacent normal gastric tissue, *H-meta*: hepatic metastasis tissue.

**Table 2:** Association between *GPR155* mRNA expression levels and clinicopathological characteristics of 200 patients with gastric cancer.

Variables	Low *GPR155* in GC tissue	High *GPR155* in GC tissue	*P*
Age			
<65 years	25	54	0.021
≥65 years	58	63	
Sex			
Male	66	75	0.017
Female	17	42	
CEA (ng/ml)			
≤5	62	93	0.426
>5	21	24	
CA19-9 (IU/ml)			
≤37	60	96	0.103
>37	23	21	
Tumor location			
Entire	7	12	
Upper third	19	25	0.972
Middle third	24	33	
Lower third	33	47	
Tumor size (mm)			
<50	26	47	0.199
≥50	57	70	
Macroscopic type			
Borrmann type 4/5	9	26	0.033
Others	74	91	
Tumor depth (UICC)			
pT1-3	47	47	0.021
pT4	36	70	
Differentiation			
Differentiated	39	35	0.014
Undifferentiated	44	82	
Lymphatic involvement			
Absent	12	14	0.607
Present	71	103	
Vessel invasion			
Absent	33	40	0.421
Present	50	77	
Infiltrative growth type			
Invasive growth	22	56	0.002
Expansive growth	61	61	
Lymph node metastasis			
Absent	22	35	0.598
Present	61	82	
Peritoneal lavage cytology			
Negative	65	80	0.118
Positive	18	37	
Synchronous hematogenous metastasis			
Absent	71	114	0.002
Present	12	3	
Synchronous hepatic metastasis			
Absent	75	114	0.031
Present	8	3	
UICC stage			
I	14	18	
II	12	21	0.916
III	26	34	
IV	31	44	
Post-operative chemotherapy (Stage II/III)			
S-1 adjuvant	21	29	0.809
None	17	26	

^*^Statistically significant (*P* < 0.05). *Abbreviations: CEA*, carcinoembryonic antigen; *CA19-9*, carbohydrate antigen 19-9; *UICC,* Union for International Cancer Control.

**Table 3:** Predictive factors for hematogenous metastasis/recurrence in 200 patients with gastric cancer.

		Hem/Hem-rec (−)	Hem/Hem-rec (+)	Univariate		Multivariable		
Variables				HR	*P*	HR	95%CI	*P*
Age	<65 years	73	6	1.59	0.352			
	≥65 years	107	14					
Sex	Male	123	18	4.17	0.027	2.42	0.55–17.3	0.259
	Female	57	2					
CEA	≤5 ng/ml	144	11	3.27	0.018	3.26	1.03–10.6	0.044*
	>5 ng/ml	36	9					
CA19–9	≤37 IU/ml	143	13	2.08	0.159			
	>37 IU/ml	37	7					
Tumor location	Lower third	71	9	0.8	0.632			
	Others	109	11					
Tumor size	<50 mm	68	5	1.82	0.248			
	≥50 mm	112	15					
Macroscopic type	Borrmann type 4/5	35	1	4.59	0.07			
	Others	145	20					
Tumor depth	pT1-3	82	12	1.79	0.219			
	pT4	98	8					
Differentiation	Differentiated	63	9	2.27	0.085			
	Undifferentiated	117	11					
Lymphatic involvement	Absent	25	1	3.06	0.212			
	Present	155	19					
Vessel invasion	Absent	72	1	12.7	<0.001	19.2	3.53–362	<0.001*
	Present	108	19					
Infiltrative growth	Invasive	77	1	14.2	<0.001	7.79	1.32–150	0.021*
	Expansive	103	19					
Lymph node metastasis	Absent	54	3	2.43	0.136			
	Present	126	17					
Peritoneal lavage cytology	Negative	127	18	3.76	0.044	3.62	0.77–27.0	0.108
	Positive	53	2					
*GPR155* expression	Low	68	15	4.94	0.001	4.24	1.40–14.8	0.010*
	High	112	5					

^*^Statistically significant in multivariate analysis (*P* < 0.05). *Abbreviations*: Hem/Hem-rec, hematogenous metastasis/recurrence; *HR*, hazard ratio; *CI*, confidence interval; *CEA*, carcinoembryonic antigen; *CA19-9*, carbohydrate antigen 19-9; *UICC,* Union for International Cancer Control.

**Table 4:** Sequences of Primers and siRNAs.

Gene	Experiment	Type	Sequence	Product size
GPR155	qRT-PCR	forward	5′-AGCAAAGCTGGACTATTCCCT-3′	125 bp
		reverse	5′-GCCACCAAATAAATGTACTGGA-3′	
	Bisulfite	forward	5′-TTTTTGTTTTTGTTTTTTAGGTTTG-3′	197 bp
	sequencing	reverse	5′-AACTAAAAATAACAATTCTATCTCC-3′	
	Knockdown	siRNA	5′-ACAUCAUAAGAGAUAUUGG-3′	
			5′-GUACAAUAGAACAAACACC-3′	
			5′-AUCAAAACUAACAUUCUGG-3′	
			5′-GUAUAUCACAGCUUCACCA-3′	
GAPDH	qRT-PCR	forward	5′-GAAGGTGAAGGTCGGAGTC-3′	226 bp
		probe	5′-CAAGCTTCCCGTTCTCAGCC-3′	
		reverse	5′-GAAGATGGTGATGGGATTTC-3′	

*Abbreviations: qRT-PCR*: quantitative real-time reverse-transcription polymerase chain reaction; *siRNA*: small interfering RNA, *bp*: base pair.
