# Supplementary material for: GPR155 Serves as a Predictive Biomarker for Hematogenous Metastasis in Patients with Gastric Cancer
Source: Sci Rep. 2017 Feb 6;7:42089. doi: 10.1038/srep42089 (PMC5292715; doi:10.1038/srep42089)
Supplement: Supplementary Information [file srep42089-s1.pdf]

Title: GPR155 Serves as a Predictive Biomarker for Hematogenous Metastasis in Patients with Gastric Cancer

Dai Shimizu, Mitsuro Kanda, Haruyoshi Tanaka, Daisuke Kobayashi, Chie Tanaka, Masamichi Hayashi, Naoki Iwata, Yukiko Niwa, Hideki Takami, Suguru Yamada, Tsutomu Fujii, Goro Nakayama, Michitaka Fujiwara, Yasuhiro Kodera

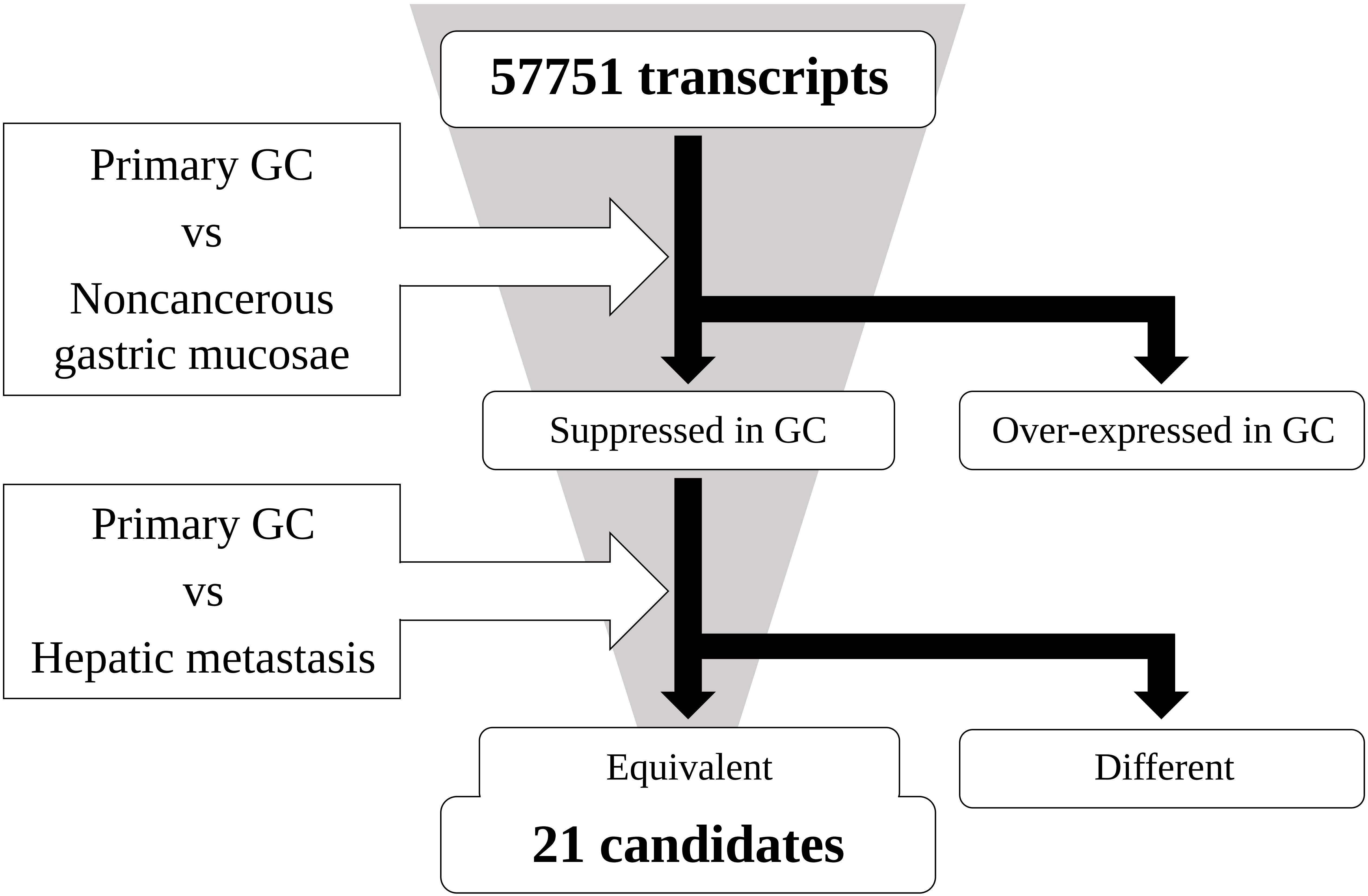

Figure S1: Criteria for identification of a candidate molecules.

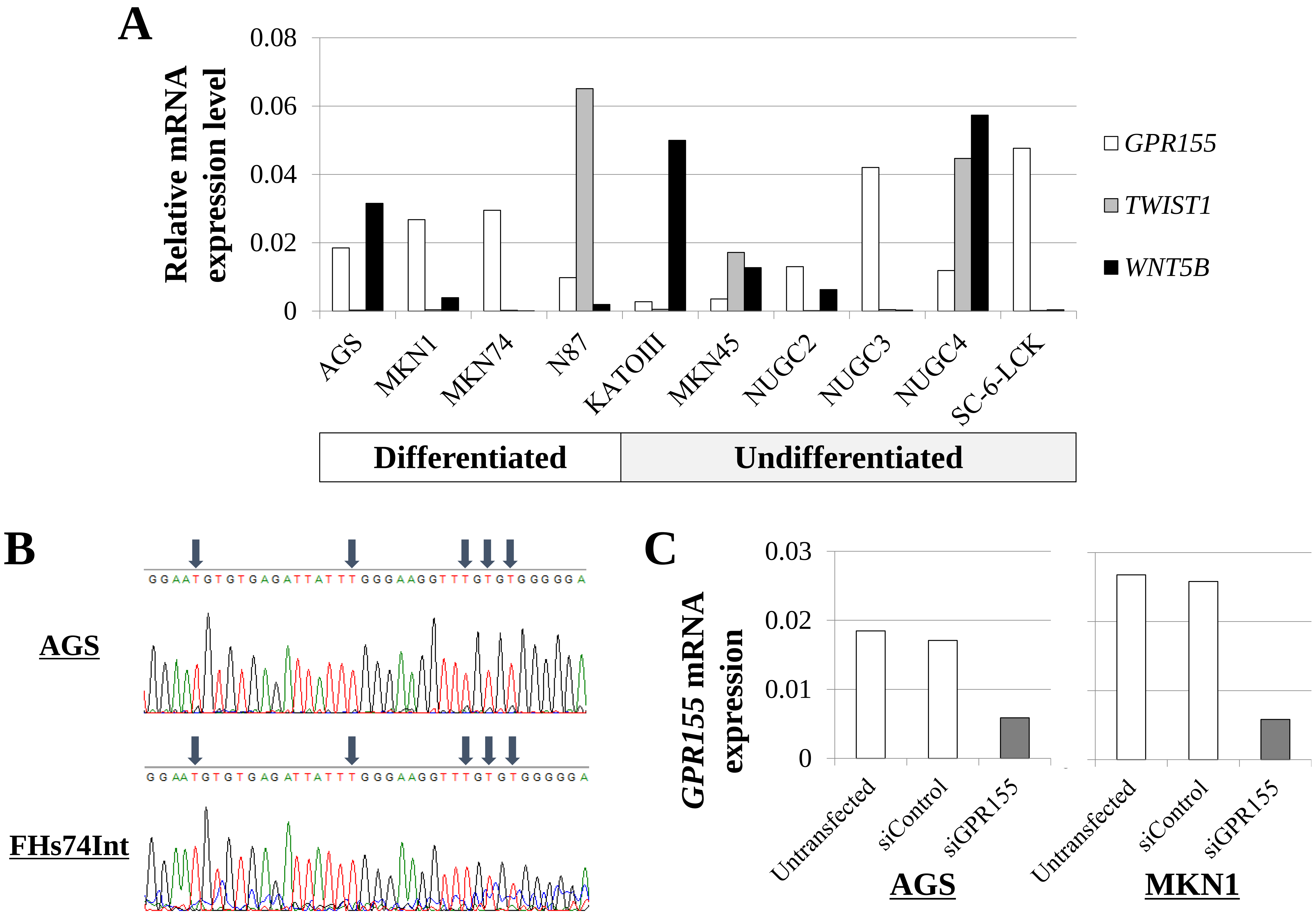

Figure S2: (A) Results of PCR array of GC cell lines. (B) Bisulfite sequence analysis of AGS and FHs74Int cells. (C) Confirmation of GPR155 mRNA knockdown by siGPR155.

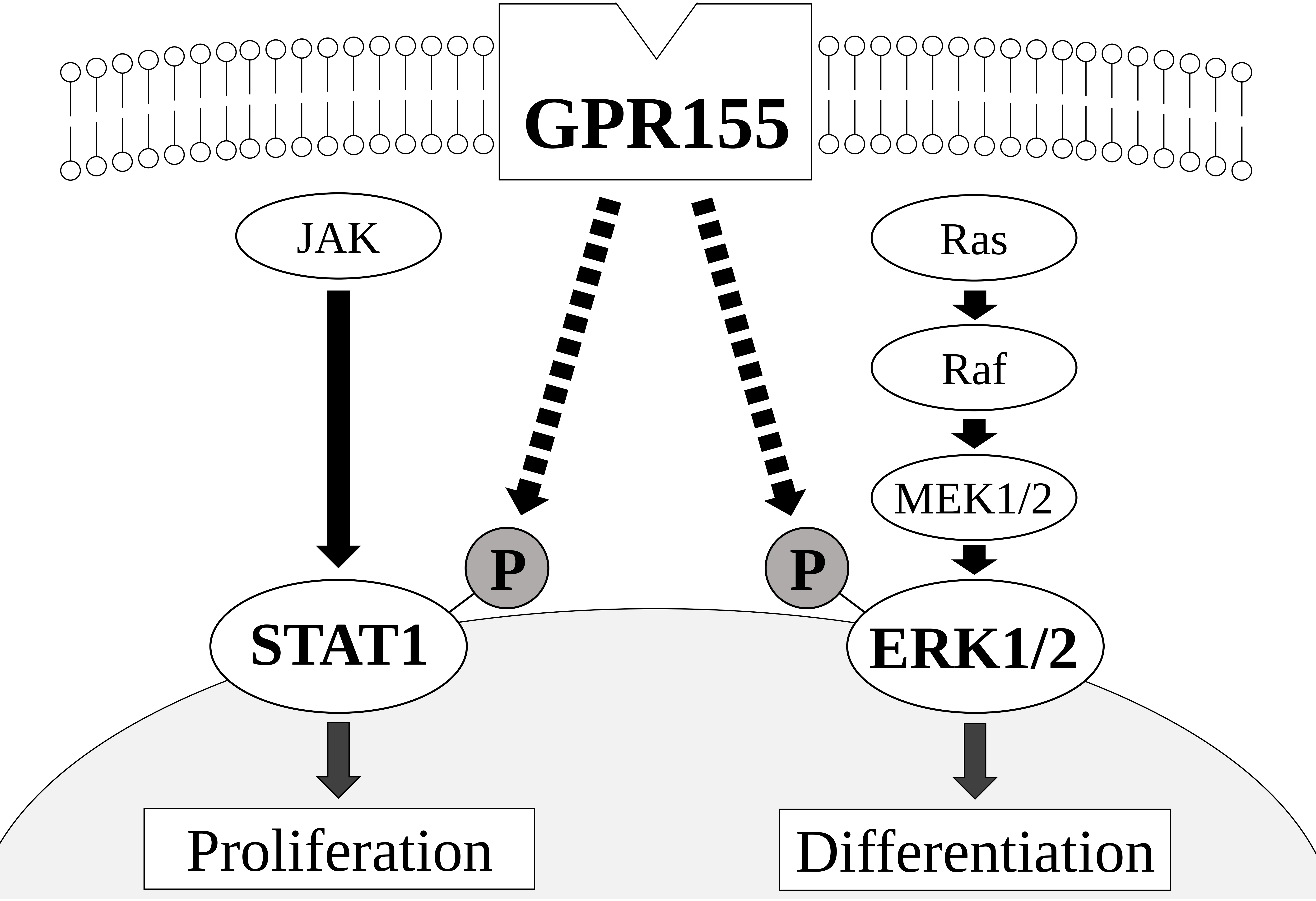

Figure S3: Schema of downstream effect of GPR155 stimulation.

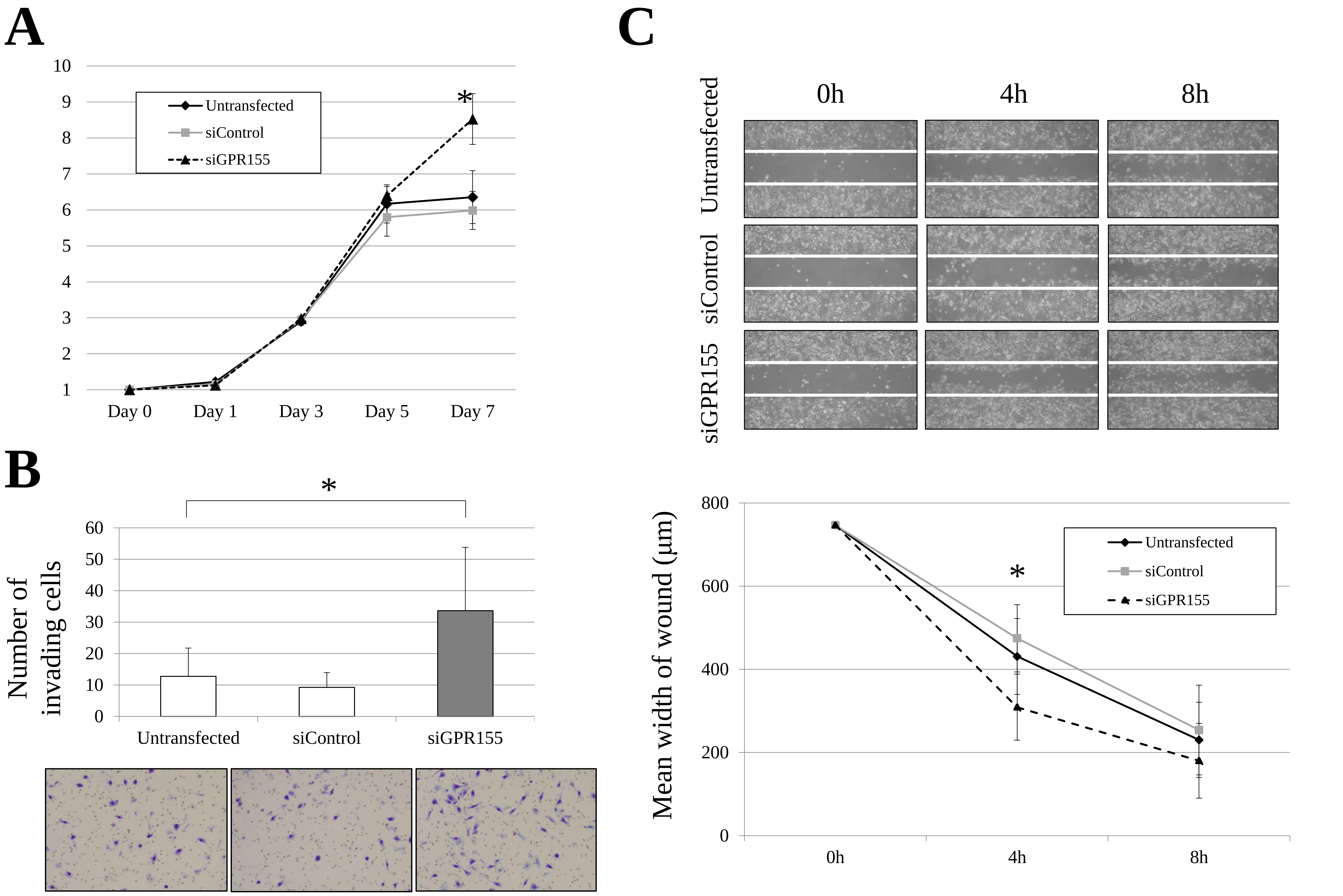

Figure S4: Phenotypes of MKN1 cells transfected with si*GPR155*.
